# Ultrasound Controllable Release of Proteolysis Targeting Chimeras for Triple-Negative Breast Cancer Treatment

**DOI:** 10.34133/bmr.0064

**Published:** 2024-08-13

**Authors:** Hongye He, Feng Li, Rui Tang, Nianhong Wu, Ying Zhou, Yuting Cao, Can Wang, Li Wan, Yang Zhou, Hua Zhuang, Pan Li

**Affiliations:** ^1^ Institute of Ultrasound Imaging & Department of Ultrasound, The Second Affiliated Hospital of Chongqing Medical University, Chongqing Key Laboratory of Ultrasound Molecular Imaging, Chongqing 400010, China.; ^2^ Department of Hepatobiliary and Pancreatic Surgery, The First Affiliated Hospital of Chongqing Medical University, Chongqing 400016, China.; ^3^ Department of Ultrasound, The Ninth People’s Hospital of Chongqing, Chongqing 400700, China.; ^4^ Department of Geriatrics, The Second Affiliated Hospital of Chongqing Medical University, Chongqing 400010, China.; ^5^ Department of Ultrasound, The Third People’s Hospital of Chengdu City, The Affiliated Hospital of Southwest Jiaotong University, Chengdu 610014, China.; ^6^ Department of Medical Ultrasound, West China Hospital of Sichuan University, Chengdu 610041, China.

## Abstract

Triple-negative breast cancer (TNBC) is a special subtype of breast cancer, which is highly aggressive and incurable. Here, we proposed an ultrasound activatable bromodomain-containing protein 4 (BRD4) proteolysis targeting chimera (PROTAC) release strategy for the first time for precisely controlled protein degradation in preclinical TNBC model. Through combination of PROTAC and ultrasound-targeted microbubble destruction (UTMD) technology, the present strategy also aims to concurrently solve the major limitations of poor loading capacity of microbubbles and undesirable targeting and membrane permeability of PROTAC. PROTAC (ARV-825)-encapsulated microbubbles, ARV-MBs, were developed for the efficacious treatment of TNBC in vitro and in vivo. The microbubbles we synthesized showed ultrasound-responsive drug release ability, which could effectively promote the penetration of PROTAC into tumor site and tumor cell. Under ultrasound, ARV-MBs could play an effective antitumor effect by potentiating the ubiquitination and degradation of BRD4 in tumor. The current study may provide a new idea for promoting clinical translation of drug-loaded microbubbles and PROTAC, and offer a new efficacious therapeutic modality for TNBC.

## Introduction

Breast cancer, the predominant malignancy among women, poses a substantial threat to national health [1]. According to the latest American Cancer Society report, breast cancer accounts for 32% of all new diagnoses in 2024 and ranks as the first-leading cause of cancer-related deaths in woman [2]. Triple-negative breast cancer (TNBC), constituting 15% to 20% of all breast cancers, emerges as the most aggressive subtype [3]. Patients diagnosed with TNBC face a median survival time of merely 12 to 18 months, with an exceedingly grim prognosis for those with advanced TNBC. Despite chemotherapy being the primary systemic therapy, the absence of a standardized optimal chemotherapy regimen exacerbates the challenges [4]. The unique molecular phenotype of TNBC renders it insensitive to conventional treatments like endocrine or molecular-targeted therapies [3], underscoring the pressing need for innovative therapeutic alternatives.

Promising preclinical agents, such as those targeting bromo and extra terminal domain proteins (BET), including JQ1, currently in clinical development, show potential [5]. These agents target the BET protein bromodomain-containing protein 4 (BRD4), disrupting its binding with acetylated chromatin and reducing the expression of critical oncogenic transcription factors like c-Myc, BCL-2, and Forkhead box M1 [6–8]. However, as with most therapies, resistance of BET inhibitors (BETis) is expected to emerge over prolonged treatment durations, diminishing therapeutic efficacy [9]. Mechanisms contributing to resistance involve the presence of a stem cell-like phenotype, activation of polo-like kinase 1, and basal activity of intracellular signaling kinases like protein kinase B or cyclin-dependent kinase activating kinase [5,10]. Overcoming this resistance is pivotal for the success of BETi-based therapies.

Proteolysis targeting chimeric (PROTAC) molecules, a novel compound family, demonstrate the ability to bind protein of interest (POI) and recruit ubiquitin ligase, promoting targeted protein degradation via the proteasome [11]. Unlike small-molecule inhibitors blocking POI’s activity, PROTAC harnesses E3-ubiquitin ligase for effective degradation, circumventing acquired drug resistance from target overexpression or mutation [12]. Unique catalytic properties endow PROTAC with advantages, including repeatability, low administration doses, and the ability to degrade undruggable proteins [13,14]. Over 20 PROTACs have entered clinical trials in recent years, and the most advanced PROTAC ARV-471, a PROTAC drug selectively degrading estrogen receptors, is now in phase III clinical trials (NCT05654623) [15], indicating the strategy’s promising potential in translating to therapeutic outcomes. However, despite these accomplishments, deficiencies such as poor aqueous solubility, limited bioavailability, tumor penetration, and cell membrane permeability hinder the extensive application of PROTACs [16]. Concurrently, systemic toxicity resulting from undesirable PROTAC activity in noncancerous tissues remains a critical safety concern [17], necessitating strategies for tumor-specific PROTAC activity.

Several ligand modification strategies, including antibody–PROTACs [18], folate-caged PROTACs [19], and aptamer–PROTAC conjugates [20], have been explored for tumor-targeted PROTAC delivery. The modified PROTACs exhibit improved cellular uptake, or enhanced tumor accumulation and subsequent augmented antitumor potency compared to conventional PROTACs. However, these complicated ligand–PROTAC modifications also bring new challenges such as low serum stability, limited tumor penetration, and heterogeneous receptor expression in different tumor cells and types [21,22]. Additionally, photo-PROTACs, developed for light-inducible protein degradation, exhibit spatiotemporal precision in vitro [23]. Yet, clinical translation of them is greatly hindered by the poor penetration depth and potential genotoxicity of commonly used ultraviolet (UV) light [24]. Therefore, precise PROTAC delivery to tumors and efficient degradation of target proteins inside tumor cells remain a formidable challenge.

Ultrasound-targeted microbubble destruction (UTMD) emerges as a safe, noninvasive strategy to enhance gene and drug delivery [25]. Microbubbles (MBs), acting as vehicles triggered by ultrasound, release drugs at the treatment site. Simultaneously, they serve as clinical contrast agents for contrast-enhanced ultrasound (CEUS) imaging [26], enabling continuous monitoring and precise control of drug delivery. Most importantly, acoustic cavitation and sonoporation resulting from UTMD can enhance tissue and cell membrane permeability, facilitating drug penetration and absorption [27]. Nevertheless, microbubbles suffer from a poor drug loading capacity due to their inherent structure and properties, which severely impedes the clinical transformation of UTMD-mediated drug delivery system [28]. Loading highly active drugs into microbubbles proves to be an effective strategy to circumvent this problem [29]. Fortunately, PROTACs, potent agents capable of degrading tumor-associated proteins at low dosages with a long duration, may serve as a perfect candidate for developing drug-loaded microbubbles. Consequently, concisely combining PROTACs with UTMD technologies is expected to be a mutually reinforcing and efficient drug delivery system with excellent clinical prospect.

**Fig. 1.**
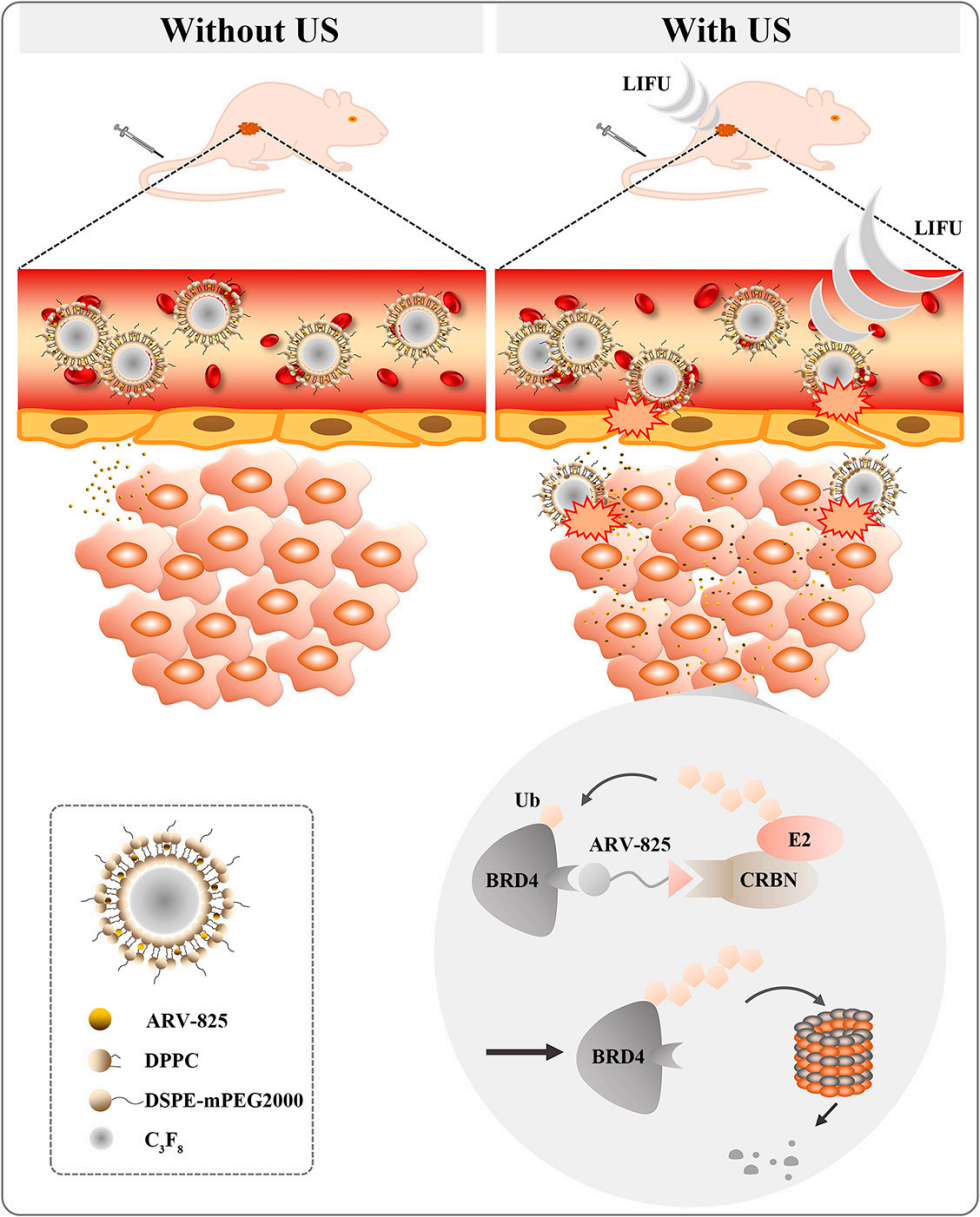
Schematic illustration of the structure model of ARV-MBs and ultrasound (US) activation mechanism of it for spatiotemporal control over PROTAC in TNBC tumor-bearing mice. Low-intensity focused ultrasound (LIFU) is a noninvasive technique using converging ultrasound waves to enhance drug delivery.

In this context, we rationally engineered a novel PROTAC formulation (ARV-825-loaded microbubbles [ARV-MBs]) for the tumor-targeted degradation of the BET protein BRD4 in TNBC. Being encapsulated into microbubbles ensures the initial silence of PROTAC’s proteolytic activity. Upon systemic administration, ARV-MBs accumulate in tumor tissues under ultrasound irradiation, leading to the enrichment of PROTAC. Moreover, the cellular uptake of drug was markedly improved because of the sonoporation effect of UTMD. Eventually, the released PROTAC restores its activity to recruit E3 ligase for efficient target protein degradation (Fig. 1). The purpose of this proof-of-principle study is to explore an efficient and clinically translatable drug delivery system based on UTMD technology for potentiated cancer therapy.

## Materials and Methods

### Preparation and characterization of ARV-MBs

ARV-MBs were prepared by a membrane hydration method combined with mechanical oscillation. Briefly, 1,2-dipalmitoyl-sn-glycero-3-phosphocholine (DPPC, 20 mg), 1,2-distearoyl-sn-glycero-3-phosphoethanolamine-N-[methoxy(polyethylene glycol)-2000 (DSPE-mPEG2000, 8 mg), and ARV-825 (4 mg) were dissolved in 10 ml of trichloromethane and the solvent was removed by rotary vacuum evaporation at 55 °C for 1 h. Next, 2 ml of glycerol phosphate-buffered saline (PBS) solution (1:9, v:v) was added to the resulting lipid film, which was hydrated via bath sonication. Afterward, the obtained liposomal emulsion was aliquoted at 500 μl to a vial. The air in the vial was replaced with perfluoropropane (C_3_F_8_) gas using syringes with 3-way stopcock. Subsequently, the bottle filled with lipid suspension and C_3_F_8_ gas was fixed in the mechanical arm of a dental amalgamator (Shanghai, China) for violent and reciprocating vibration at a high frequency (~3,200 rpm). The free drug (not incorporated into the microbubbles) was discarded by centrifugation (300*g*, 3 min) to obtain purified ARV-MBs. The unloaded microbubbles (non-drug-loaded) were similarly prepared but without the addition of ARV-825.

Digital photographs of unloaded MBs and ARV-MBs were taken. The morphology of these microbubbles was observed under a confocal laser scanning microscopy (CLSM; Andor, UK). The size and potential of microbubbles were measured by Malvern particle size potentiometer. The loading efficiency and release profile under ultrasound irradiation of ARV-825 in the microbubbles were determined by UV-visible (UV-vis) spectrophotometry using a UV-2550 spectrophotometer (Shimadzu, Japan).

### Animal model

All mouse experiments were performed in accordance with protocols approved by the Institutional Animal Care and Use Committee at Chongqing Medical University. Balb/c nude female mice (4 to 5 weeks old) were purchased from Changzhou Cavins Laboratory Animal Co. Ltd. (license no. SCXK (Su) 2021-0013). TNBC tumor model was established by subcutaneous injection of MDA-MB-231 cells (2 × 10^6^) suspended in a mixture of Matrigel in PBS.

### Intracellular and intratumoral ARV-825 concentration

MDA-MB-231 cells were plated in a 10-cm cell culture dish and incubated for 24 h. ARV-MBs were added with or without ultrasound irradiation (1.0 MHz, 30% duty ratio, 1 W/cm^2^, 30 s). After 2 h, the cells were collected via centrifugation and suspended with PBS. Then cells were lysed using cell lysis solution to release intracellular drugs. Samples were centrifuged at 15,000 rpm for 10 min, and the supernatant was analyzed with reversed-phase high-performance liquid chromatography (RP-HPLC) to detect ARV-825 concentration.

Tumor-bearing mice were randomly divided into 2 groups (*n* = 3). One group was injected with ARV-MBs (containing ARV-825 3.5 mg/kg, 200 μl) through the tail vein, and the other group was injected with the same amount of ARV-MBs and immediately irradiated with ultrasound (1.0 MHz, 30% duty ratio, 2 W/cm^2^, 3 min) after injection. One hour later, the tumor tissues were collected, weighed, and homogenized. The pre-cooled methanol was added, and the supernatant was obtained after centrifugation at 15,000 rpm for 10 min (4°C). The content of ARV-825 in tumor tissues was analyzed by RP-HPLC.

### In vivo tumor growth inhibition

When the tumor volume reached about 60 mm^3^, the mice were randomly divided into 6 groups: control (C), ultrasound (US), unloaded microbubbles + ultrasound (MBs + US), drug-loaded microbubbles (ARV-MBs), free drug (ARV), and drug-loaded microbubbles + ultrasound (ARV-MBs + US). The mice were intraperitoneally injected with 10 mg/kg ARV-825 or intravenously injected with 200 μl of saline, microbubbles, or ARV-MBs (ARV-825: 3.5 mg/kg). After injection, the tumor of each mouse was treated with or without ultrasound (1.0 MHz, 2.0 W/cm^2^, 30% duty ratio, 3 min) immediately. During the ultrasound process, the thickness of ultrasonic couplant was fixed and the probe was moved to ensure that the whole tumor could be irradiated. Treatment was performed every other day for a total of 5 times. The body weight of mice and size of tumors were measured every other day for 14 days. The tumor volume was calculated according to the following formula: volume = [tumor length × tumor width^2^]/2. The mice were executed at day 14. The tumors were collected for hematoxylin and eosin (H&E) staining, proliferating cell nuclear antigen (PCNA) immunohistochemical staining, terminal deoxynucleotidyl transferase–mediated deoxyuridine triphosphate nick end labeling (TUNEL) immunofluorescence staining, as well as molecular expression analysis.

### Co-immunocoprecipitation

The cells were seeded in 10-cm dish and transfected with pcDNA3.1 (vector) or Flag-CRBN plasmid for 48 h. Then, the cells were treated with vehicle, ARV-825, or ARV-MBs with or without ultrasound (1.0 W/cm^2^, 30 s, 1.0 MHz, 30% duty ratio) for 24 h. Afterward, MG132 with a final concentration of 10 μM was added to each dish 6 h before harvest. The cells were lysed with IP lysis buffer containing 1% protease inhibitor for 20 min on ice and centrifuged at 12,000 rpm for 15 min at 4°C. One-fifth of supernatant was used as input, and the remnant lysates were used for pulldown. The samples were incubated with anti-Flag primary antibody and agarose beads at 4°C overnight. After washing 4 times with PBS, the immunoprecipitates were resuspended in 1 × loading buffer and boiled at 100 °C for 5 min. The protein samples were then analyzed by Western blot.

### The ubiquitination of BRD4

The cells were transfected with hemagglutinin-tagged ubiquitin (HA-Ub) plasmid together with pcDNA3.1 or Flag-CRBN plasmid for 48 h. Then, the cells were treated as above and the protein samples were collected for IP assay. The ubiquitination was visualized by Western blot using anti-BRD4 antibody.

### Statistical analysis

The GraphPad Prism software was used for statistical analysis. Data were expressed with mean ± SD. The Student’s *t* test was used for 2-group comparison. The ordinary one-way analysis of variance (ANOVA) was applied for multiple groups comparisons. *P < 0.05 was considered statistically significant.

## Results

### Formulation and characterization of ARV-MBs

**Fig. 2.**
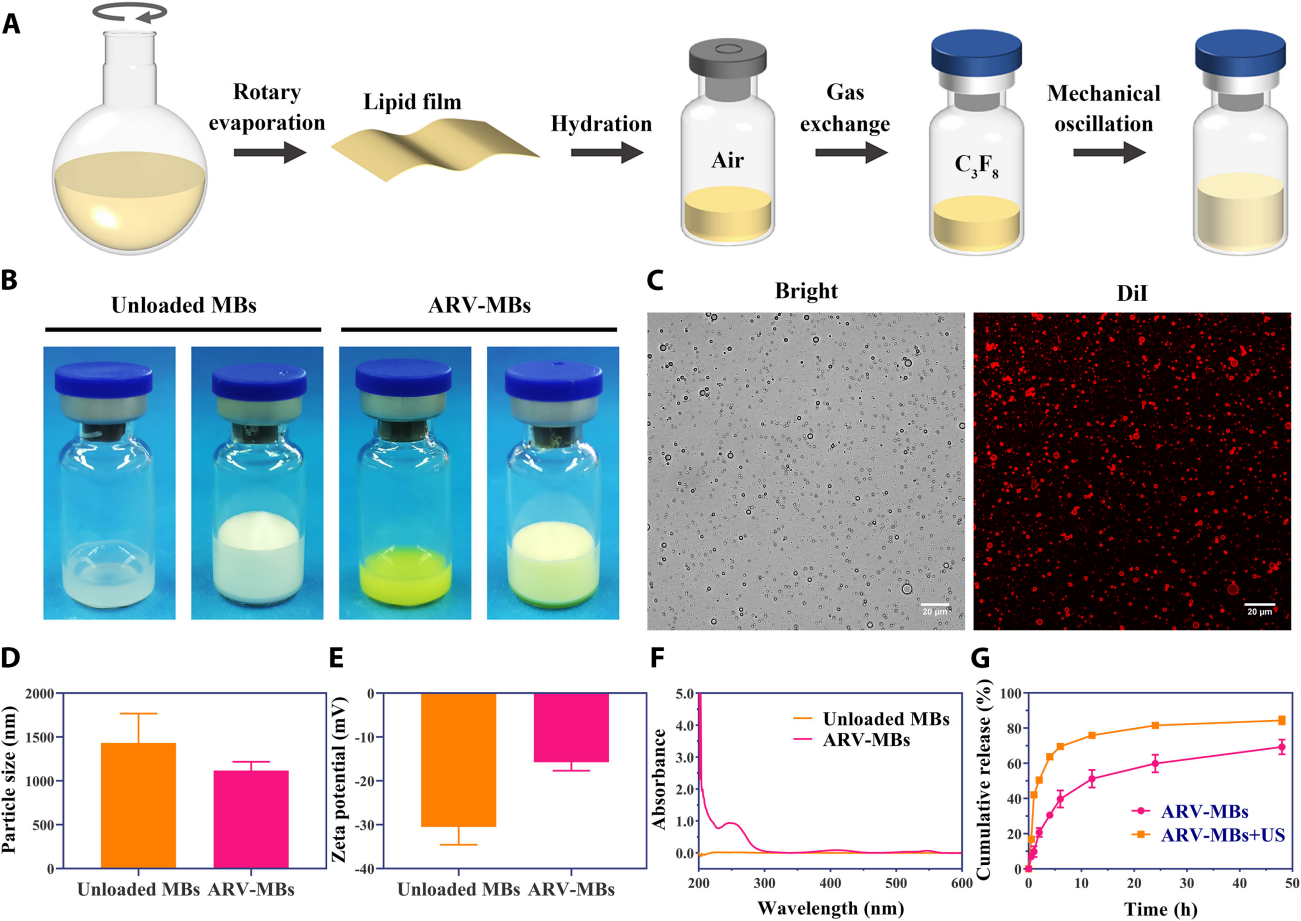
Formulation and characterization of ARV-MBs. (A) Schematic illustration of ARV-MBs synthesis. (B) Photographs of unloaded microbubbles and ARV-MBs before and after mechanical oscillation under daylight. (C) Bright-field image and fluorescence image of ARV-MBs by CLSM. Scale bar, 20 μm. (D) Size distribution of unloaded microbubbles and ARV-MBs. (E) Zeta potential of unloaded microbubbles and ARV-MBs. (F) UV-Vis absorption of unloaded microbubbles and ARV-MBs. (G) Cumulative release profiles of ARV-825 from ARV-MBs without or with ultrasound irradiation (*n* = 3).

Microbubbles carrying ARV-825 were successfully prepared by a membrane hydration method combined with mechanical oscillation (Fig. 2A). The ARV-MBs appeared as yellow suspension before oscillation, and light yellow and milky suspension after oscillation (Fig. 2B). The drug-carrying microbubbles displayed a uniform bubble shape with smooth surface and good dispersion, and DiI successfully labeled the phospholipid shell of microbubbles under CLSM (Fig. 2C). The average particle size of unloaded microbubbles was 1.43 μm, and the average potential was −30.53 mV, respectively. After drug encapsulation, the size and potential of microbubbles decreased slightly (1.12 μm and −15.75 mV) (Fig. 2D and E and Fig. S1A and B). During 6 days of storage at 4°C, the size of ARV-MBs remained relatively stable over time (Fig. S2).

UV-vis spectrophotometer analysis showed that the prepared drug-carrying microbubbles showed characteristic absorption peaks of ARV-825, which confirmed the successful preparation of ARV-MBs (Fig. 2F). The drug encapsulation rate and loading content were 70.42 ± 6.13% and 8.80 ± 0.77%, respectively (calculated with the standard curve of pure drug in Fig. S1C and D). Furthermore, as shown in Fig. 2G, the drug release of ARV-825 was induced by ultrasound excitation, which was rapid in the first and closed to the peak at 12 h, while the release of ARV-825 was relatively slow in the group without ultrasound. The possible reason for the narrowing of the difference between the 2 groups at 48 h was that most of the microbubbles were spontaneously broken after a long-time incubation.

### Internalization and penetration of ARV-MBs

**Fig. 3.**
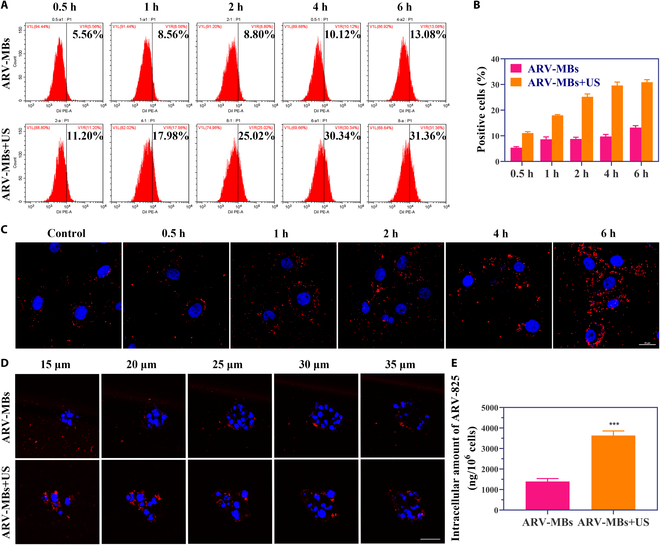
Qualitive cellular uptake assay. (A and B) Flow cytometry plots (A) and corresponding quantitative fluorescence intensity analyses (B; *n* = 3) of MDA-MB-231 cells treated with DiI-labeled microbubbles with or without ultrasound at different times. (C) Intracellular fluorescence of MDA-MB-231 cells incubated with DiI-labeled microbubbles for different times observed by CLSM. Scale bar, 25 μm. (D) Fluorescence distribution of 3D tumor spheroids incubated with ARV-MBs with or without ultrasound observed by CLSM. Scale bar, 50 μm. (E) Quantitative analysis of intracellular amount of ARV-825 in MDA-MB-231 cells treated with ARV-MBs in the presence or absence of ultrasound as determined by RP-HPLC (*n* = 3, ***P < 0.001).

In order to evaluate the effect of UTMD on drug uptake, the microbubbles were labeled with DiI fluorescent dye, and cell uptake efficiency was detected. Flow cytometry showed that the fluorescence signal increased with incubation time, suggesting that cell uptake of MDA-MB-231 cells increased with time. At the same time point, microbubbles combined with ultrasound showed stronger fluorescence signals than microbubbles alone (Fig. 3A and B), indicating that the destruction of microbubbles by ultrasound could promote cell uptake. This may be due to the cavitation effect caused by the destruction of microbubbles by ultrasound and the perforation of cell membrane surface adjacent to microbubbles, thus improving cell uptake efficiency. The results of CLSM were consistent with those of flow cytometry. At 6 h, obvious red fluorescence was observed in the cytoplasm of the ultrasound combined with the microbubbles group, while only a little fluorescence was observed around the cell membrane in the microbubbles alone control group (Fig. 3C).

Compared with 2-dimensional (2D) cell culture, 3D multicellular tumor spheroid model is recognized as one of the best models for tumor research, which is closer to the pathophysiological characteristics of tumor tissues. Thus, we used 3D tumor spheroid to further analyze the role of ultrasound-destructive microbubbles in tumor penetration. The results showed that, compared with microbubbles alone, the microbubbles with ultrasound showed red fluorescence distribution in the center of the tumor spheroid (Fig. 3D), suggesting that the combination of microbubbles and ultrasound could promote penetration of drugs deep into the tumor. In order to more directly and accurately verify the influence of UTMD on drug uptake, we collected the treated cells and quantitatively detected the drug content by RP-HPLC. Compared with the drug-carrying microbubbles alone group, ultrasound destruction of drug-carrying microbubbles increased the intracellular ARV-825 concentration (2.6 times) (Fig. 3E).

### In vivo biodistribution and tissue drug delivery

**Fig. 4.**
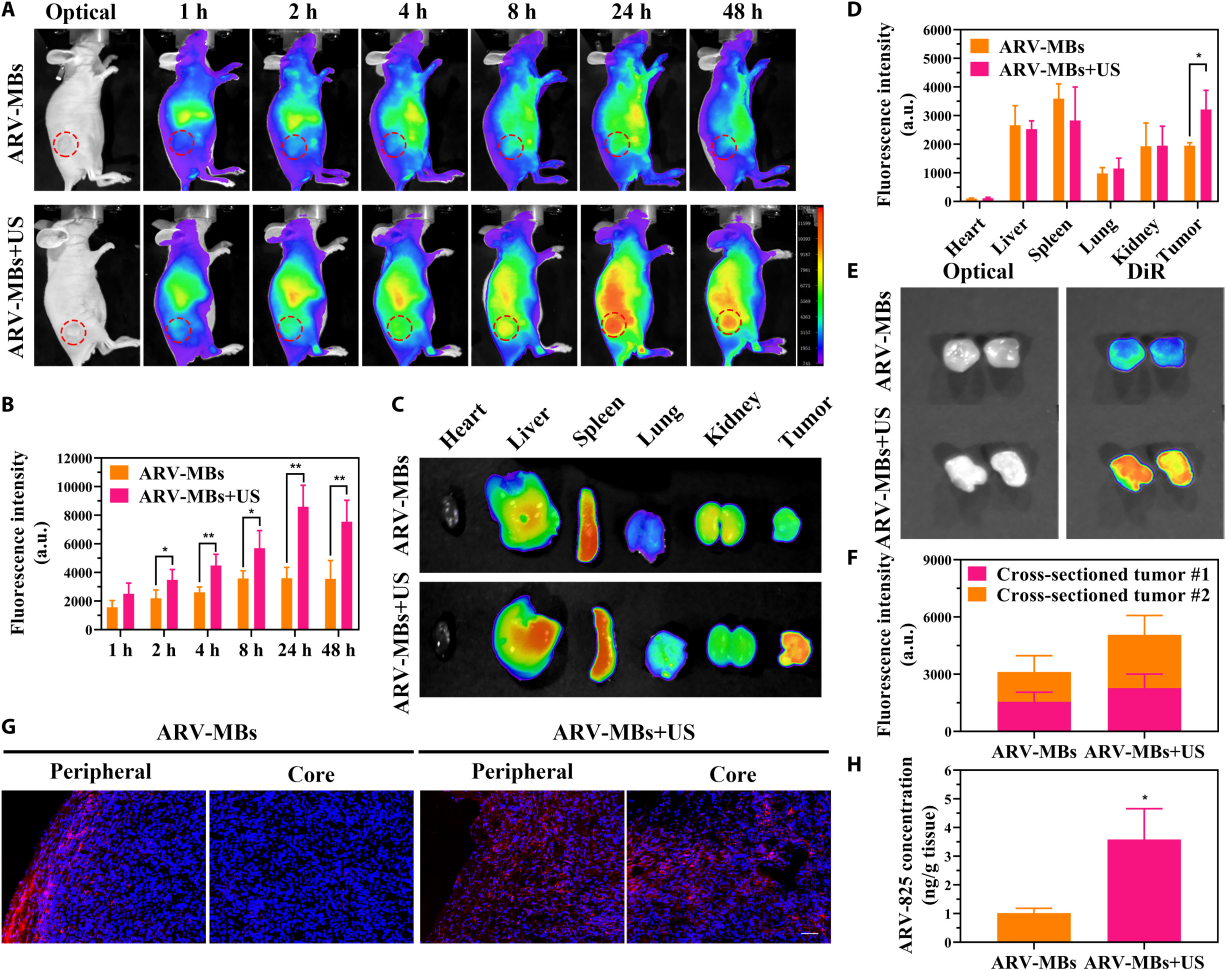
In vivo tumor accumulation of ARV-MBs amplified by UTMD. (A) Representative in vivo fluorescence images of MDA-MB-231 tumor-bearing mice at different times after intravenous injection of ARV-MBs with or without ultrasound irradiation. The red dashed circles indicate the tumor. (B) Quantification of fluorescence intensities (FIs) in tumors at different time points (*n* = 4, *P < 0.05, **P < 0.01). (C) Ex vivo fluorescence images of the vital organs (heart, liver, spleen, lung, kidney) and tumor (the vital organs and tumors were harvested 48 h after treatment). (D) Quantification of FIs in tumor and major organs from (C) (*n* = 4, *P < 0.05). (E) Ex vivo fluorescence images of the cross-section of dissected tumors. (F) Intratumoral fluorescence intensity of dissected tumors in (E). (G) Ex vivo fluorescence images of the tumor sections. Scale bar, 50 μm. (H) HPLC-determined intratumoral ARV-825 contents (*n* = 3, *P < 0.05).

In vivo fluorescence imaging of MDA-MB-231 nude mice showed that the fluorescence signal of tumor was enhanced with time in both the microbubbles group and the microbubble combined with ultrasound group, and reached a peak at 24 h. However, at the same time point, the tumor fluorescence signal of microbubble with ultrasound was significantly stronger than that of microbubbles alone (Fig. 4A and B). Ex vivo tissue fluorescence imaging further confirmed this conclusion. The fluorescence signal of the tumor in the microbubble with ultrasound group was stronger than that in microbubbles alone group, while there was no significant difference between the 2 groups in other vital organs (Fig. 4C and D). Further, obvious fluorescence distribution in the central area of tumor was exhibited in the microbubble with ultrasound group, and the overall tumor fluorescence was stronger than that in the microbubble group (Fig. 4E and F). Consistently, the results of tumor tissue sections showed that red fluorescence was mainly distributed in the periphery of the tumor in the microbubble group, and the infiltration of red fluorescence into the deep tissue was observed in the microbubble with ultrasound group (Fig. 4G), indicating that ultrasound promoted the infiltration of microbubble loads into the tumor center. More importantly, RP-HPLC quantitative analysis showed that the combination of microbubble with ultrasound gave rise to a remarkable drug accumulation in tumor tissues. The concentration of ARV-825 in tumor treated with microbubble and ultrasound was 3.5-fold higher than that of microbubbles alone (Fig. 4H).

### In vitro and in vivo CEUS

**Fig. 5.**
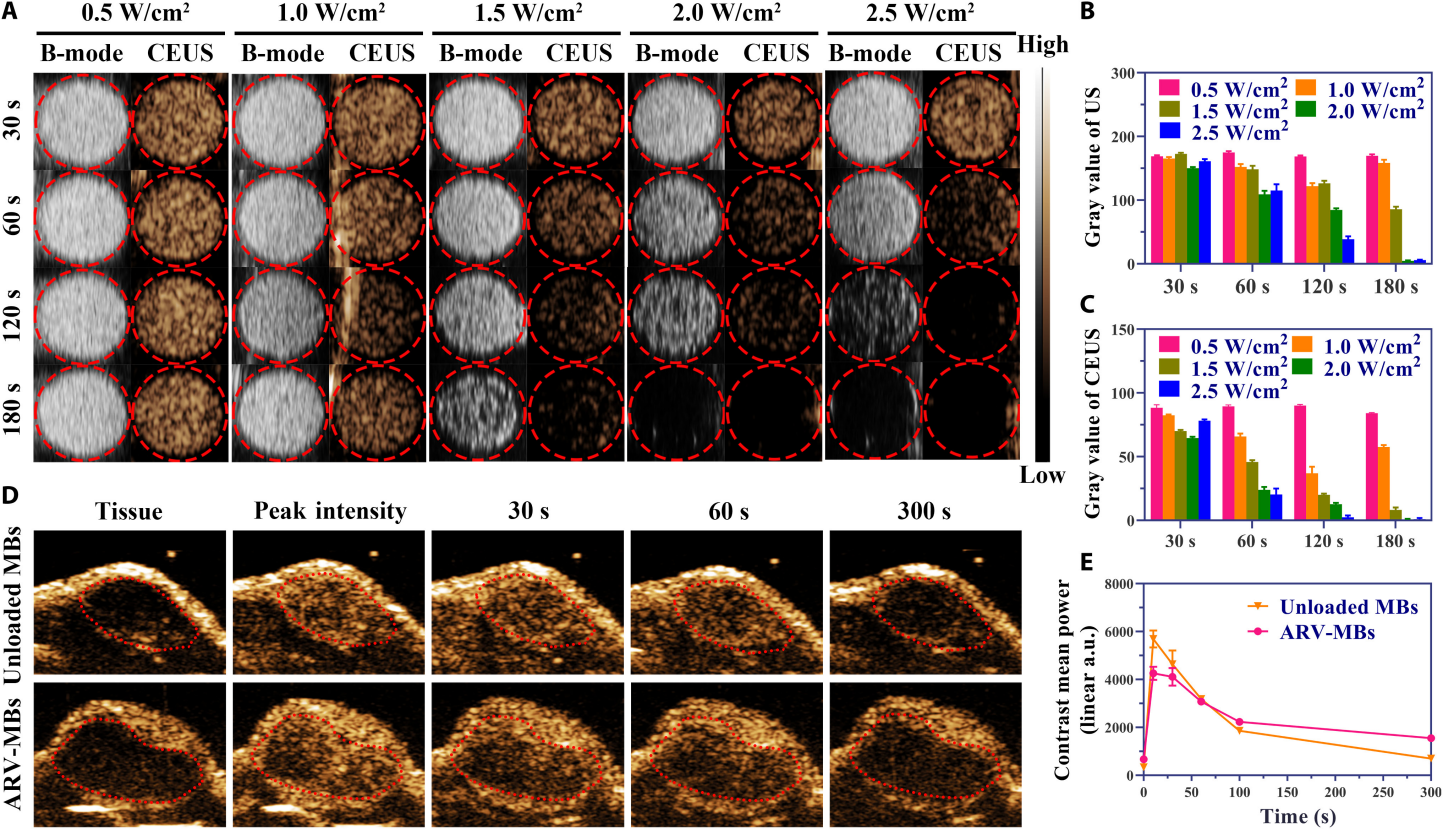
In vitro and in vivo CEUS imaging. (A) CEUS images of microbubbles treated with different ultrasound conditions in vitro. (B and C) Corresponding gray values of ultrasound (B) and CEUS (C) signals in (A). (D) Ultrasound contrast images of TNBC xenografts in mice after injection of unloaded or drug-loaded microbubbles. (E) Time–intensity curve analysis of (D).

The in vitro CEUS results showed that with the increase of ultrasonic irradiation power and the irradiation time, the contrast-enhanced signals gradually disappeared, and the signals in B mode and contrast-enhanced mode completely disappeared after 2.0 W/cm^2^ of ultrasound irradiation for 3 min (Fig. 5A to C). In in vivo experiments, contrast signal began to fill in the tumor area approximately 3 s after microbubble injection and reached the peak at about 5 s, and the signal was still present 5 min later (Fig. 5D). The time–intensity curve showed that the contrast-enhanced imaging results of drug-carrying microbubbles were similar to those of unloaded microbubbles (Fig. 5E).

### In vitro antitumor effect

**Fig. 6.**
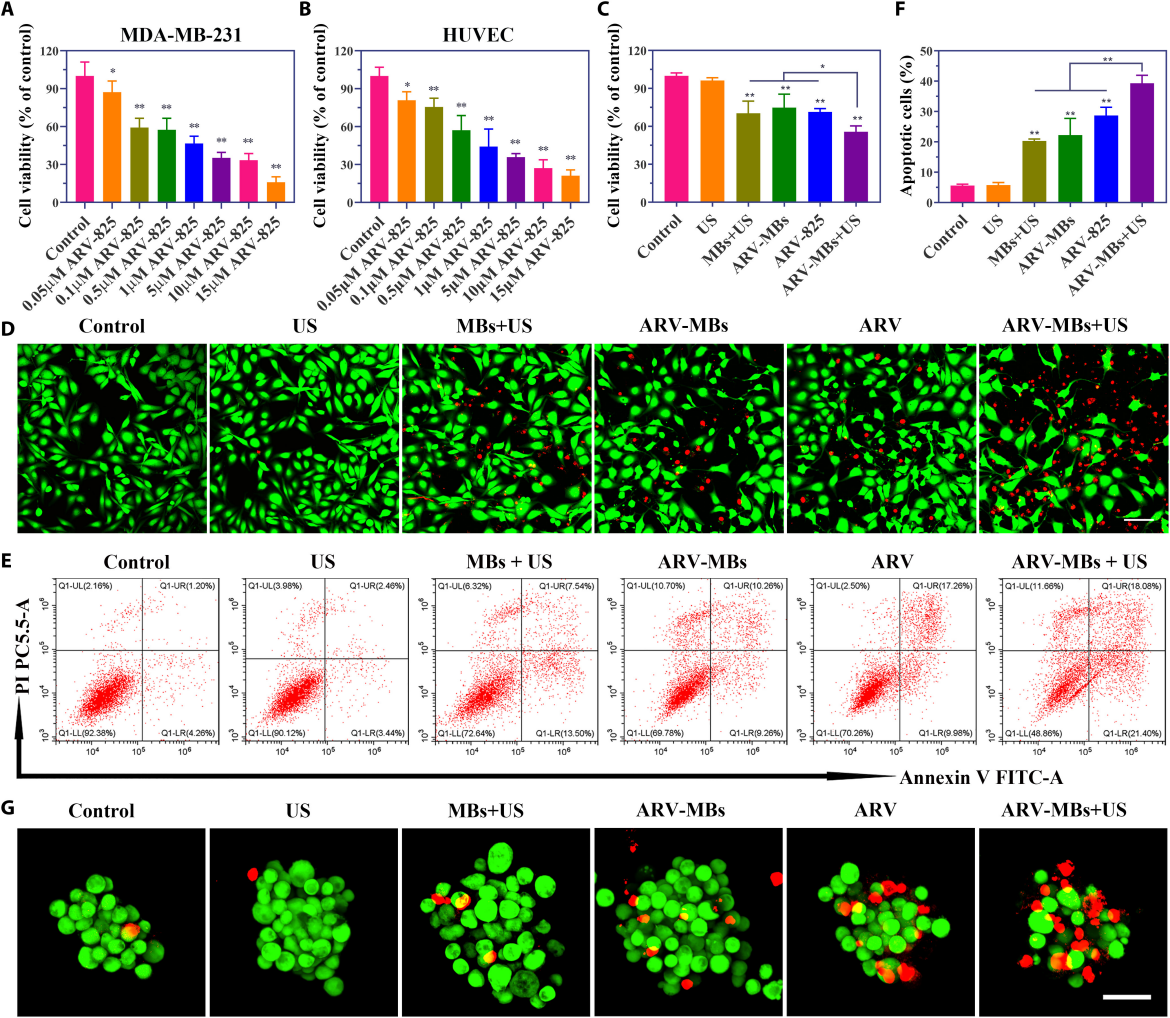
Inhibitory effects and cell apoptosis in vitro. (A and B) The cell viability assays using CCK-8 reagent were conducted in MDA-MB-231 cells (A) and HUVECs (B) incubated with ARV-825 at different concentrations for 48 h. *P < 0.05, **P < 0.01. (C) Cell viability study by CCK-8 assay in MDA-MB-231 cells after various treatment for 24 h. *P < 0.05, **P < 0.01. (D) Representative images of living and dead staining (stained with calcein-AM [green] and PI [red], respectively) of MDA-MB-231 cells after different treatments as observed by CLSM. Scale bar, 100 μm. (E) Annexin V/PI double staining detected by flow cytometry in MDA-MB-231 cells. (F) Percentages of apoptosis cells. **P < 0.01. (G) Representative images of living and dead staining by CLSM in 3D tumor spheroids after different treatments. Scale bar, 50 μm.

The cell counting kit-8 (CCK-8) results showed that ARV-825 displayed a potent antiproliferation effect in MDA-MB-231 cell lines in a concentration-dependent manner, and unfortunately, it also significantly suppressed the cell proliferation of human umbilical vein endothelial cells (HUVECs) after both 48 and 72 h of incubation (Fig. 6A and B and Fig. S3A and B). The undifferentiated inhibition of ARV-825 on normal endothelial cells highlights the necessity and rationality of controlled-release drugs by ultrasound. Further results suggested that, compared with ARV-825 and ARV-MBs, ARV-MBs + US were much more potent in suppressing cell proliferation in MDA-MB-231 cells, and the cell survival rate was the lowest among groups, preliminarily indicating that the drug-loaded microbubbles with ultrasound had the strongest in vitro antitumor effect (Fig. 6C).

In order to more intuitively show cell survival, we performed calcein-AM/propidium iodide (PI) live/dead cell staining and annexin V-fluorescein isothiocyanate (FITC)/PI apoptosis staining. Laser confocal detection of live and dead staining showed that a few dead cells with red fluorescence staining were observed in MBs + US, ARV, and ARV-MBs groups, while a large number of cells with red fluorescence were detected in the ARV-MBs + US group, and the US group showed almost all living cells with green fluorescence (Fig. 6D). This is suggested that ARV-MBs + US can cause the most potent cell death. In the apoptotic flow test, the percentage of apoptotic cells in the ARV-MBs + US group (39.31%) was significantly higher than that in the control group (5.62%), and the percentage of apoptotic cells in ARV and ARV-MBs groups was moderately increased (28.69% and 22.22%, respectively), while there was no significant difference between the US group and the control group (5.77%; Fig. 6E and F). The 3D tumor spheroid model was further used to analyze the effect of in vitro treatment, and the results showed that the drug-loaded microbubbles with ultrasound group exhibited the highest proportion of dead cells (Fig. 6G), which was consistent with the live and dead staining results observed in 2D cell culture. In summary, these results validate the capability of ARV-MBs + US to effectively inhibit TNBC cell proliferation and show its superior potency compared to native drug alone.

### Synergistic antitumor activity of ARV-MB-based UTMD in treating TNBC

**Fig. 7.**
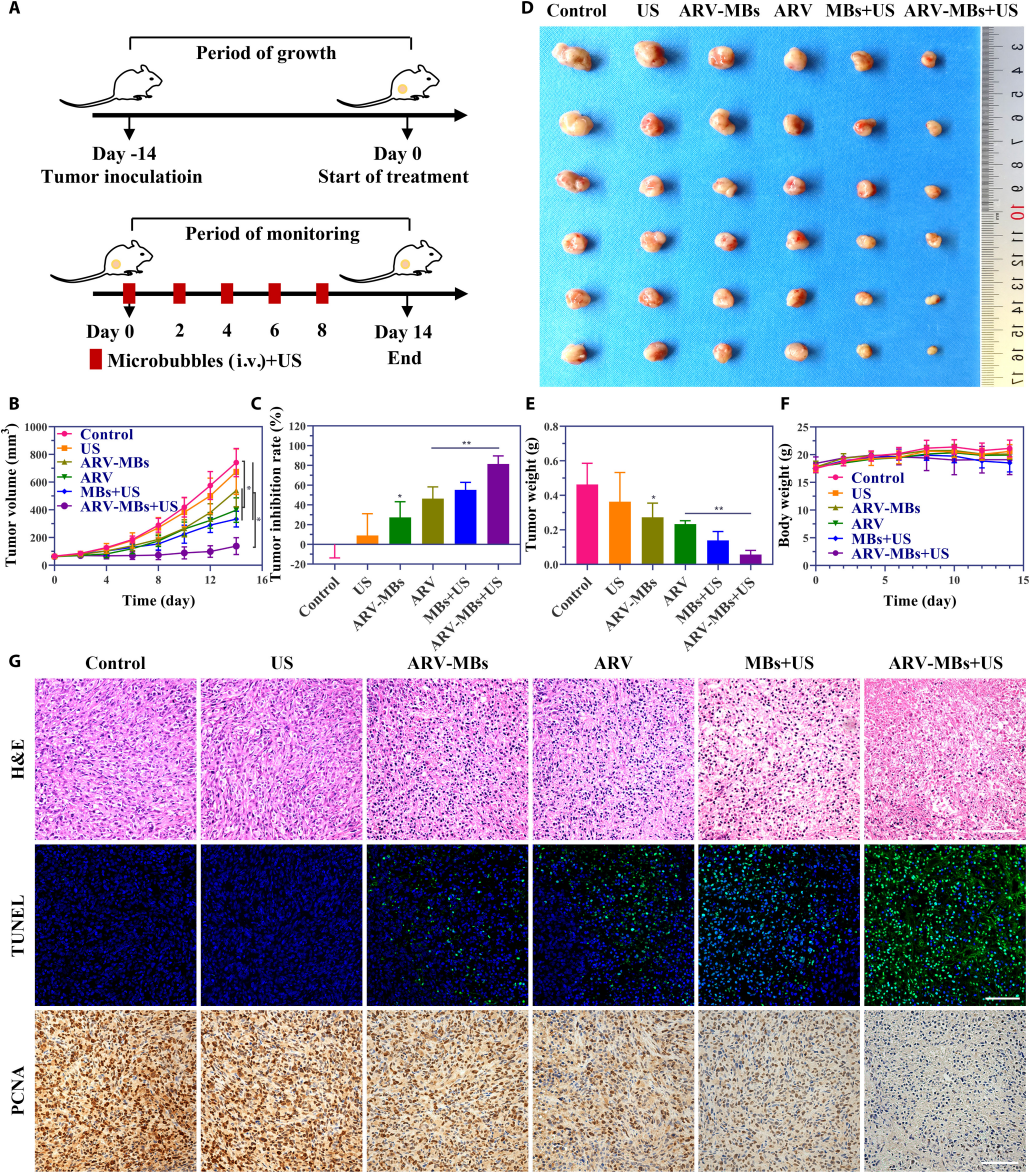
ARV-MBs with ultrasound improved antitumor activity in TNBC xenograft model. (A) Experimental schedule for antitumor study in TNBC mice model. MDA-MB-231 cells were subcutaneously injected 14 days before treatment. i.v., intravenous. (B) Tumor growth curves of different groups during 14-day monitoring. *n* = 6, *P < 0.05. (C) Tumor inhibition rates at the end of monitoring. *n* = 6, *P < 0.05, **P < 0.01. (D) Typical photographs of tumor tissues dissected at day 14 from mice subjected with different treatments. (E) Tumor weights at the end of monitoring. *n* = 6, *P < 0.05, **P < 0.01. (F) Body weight change of mice during the monitoring period. *n* = 6. (G) Representative images of H&E, TUNEL, and PCNA staining of excised tumors in various groups. Scale bars, 100 μm.

In order to further verify the in vivo antitumor effect of ultrasound destruction of drug-carrying microbubbles, we established a mouse transplanted tumor model of human TNBC (Fig. 7A). The results of tumor volume monitoring showed that no significant difference was found between the ultrasound and the control group. The tumor growth in the ARV, ARV-MBs, or MBs + US group slowed down to a certain extent. As expected, the mice in the group of ARV-MBs exposed to ultrasound manifested the slowest tumor growth and the highest tumor inhibition rate (81.4%) (Fig. 7B and C). The typical photographs and weights of excised tumors at the end of monitoring further confirmed the stronger in vivo antitumor efficacy of the ARV-MBs + US group as compared to other groups (Fig. 7D and E). During the monitoring period, there was no significant difference in body weight between different groups (Fig. 7F). Furthermore, as the histological analysis displayed in Fig. 7G, H&E staining showed that the tumor tissue structure was destroyed and the tumor cell morphology was lost in the ARV-MBs + US group. Accordingly, the expression of PCNA, a proliferation marker [30], in the ARV-MBs + US group was the lowest. TUNEL staining is an experimental method to show apoptotic cells, and positive staining indicates apoptotic cells [31]. Similar to the results of PCNA, the number of TUNEL-positive stained cells in the drug-carrying microbubbles with ultrasound group was the highest.

### The effects of ARV-MB-based UTMD on BRD4 degradation

**Fig. 8.**
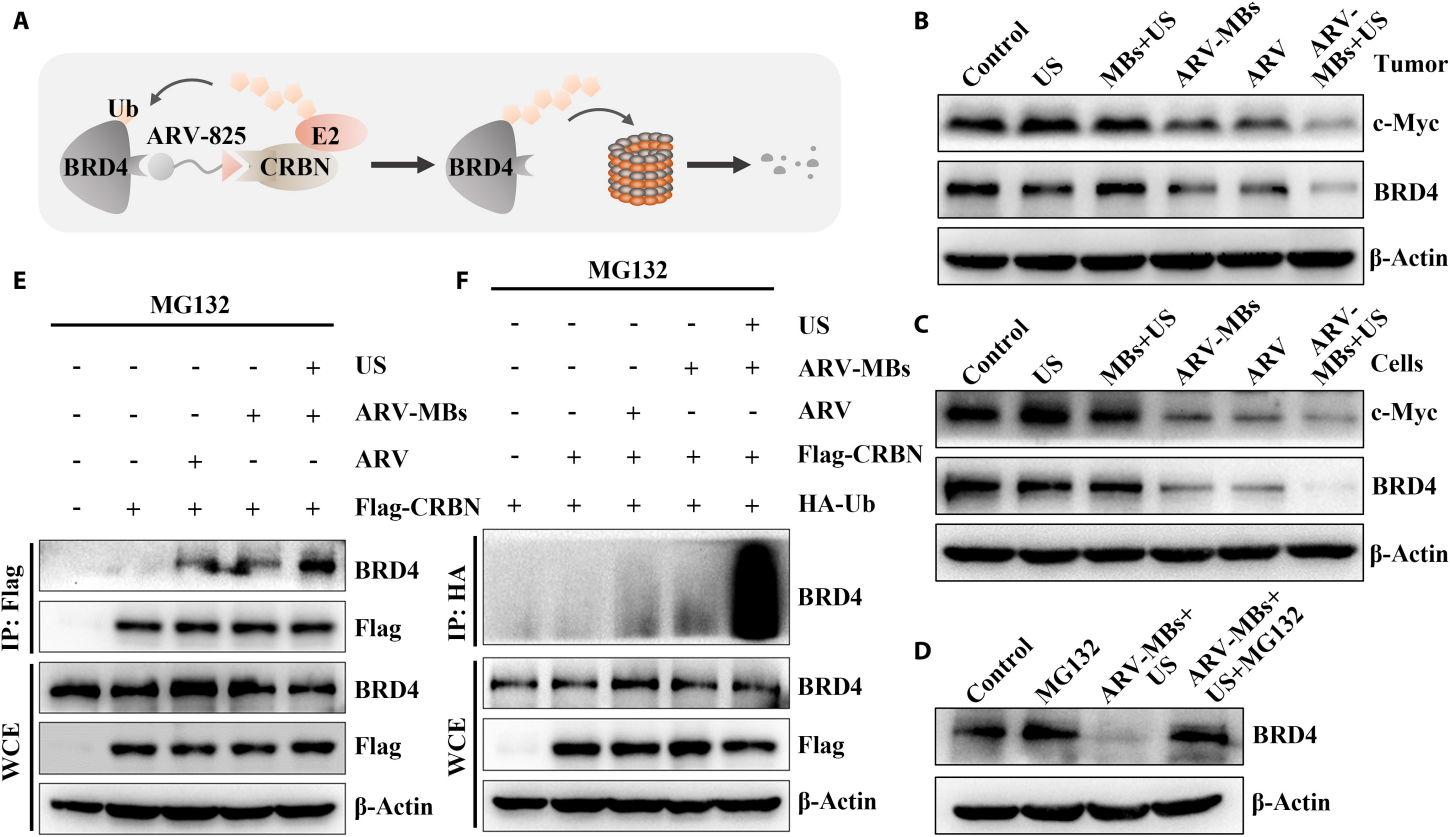
UTMD amplifies BRD4 degradation and ubiquitination induced by ARV-825. (A) Schematic illustration of ARV-825-mediated BRD4 degradation. (B) The indicated protein levels of tumor tissues were determined by Western blot (β-actin as the loading control). (C) The indicated protein levels of MDA-MB-231 cells after different treatments were determined by Western blot and normalized with β-actin. (D) Protein expression level of BRD4 in MDA-MB-231 cells after different treatments as detected by Western blot (β-actin as the loading control). (E) Interaction of BRD4 and CRBN determined by co-IP assay. (F) Ubiquitination of BRD4 detected by co-IP assay.

The key mechanism of ARV-825 in tumor inhibition is the ubiquitination degradation of BRD4 (Fig. 8A). Therefore, we first detected BRD4 protein expression in tumor tissues by Western blot. Compared with the control group, there was no obvious reduction of BRD4 protein level in US and MBs + US groups, while the ARV and ARV-MBs groups showed a moderate decrease, and the decrease was most potent in the ARV-MBs + US group. The protein expression trend of c-Myc, which was downstream target of BRD4, was consistent with that of BRD4 (Fig. 8B). Furthermore, similar to Western blot results of tumor tissues, ARV-MBs + US treatment could effectively reduce the expression of BRD4 and c-Myc in tumor cells (Fig. 8C).

The proteasome was further blocked by the proteasome inhibitor MG132 to verify whether the decreased expression of BRD4 protein was due to protein degradation through the ubiquitin proteasome system. The results showed that MG132 treatment could completely block the BRD4 protein downregulation induced by ARV-MBs + US (Fig. 8D), suggesting that ARV-MBs + US treatment could “knock out” BRD4 protein mainly by promoting the ubiquitination degradation of BRD4. It has been revealed that ARV-825 can bind BRD4 and E3 ubiquitin ligase CRBN, respectively, to narrow down the spatial distance between them, and promote ubiquitination of the former. Next, we examined the binding of BRD4 to CRBN and the ubiquitination of the substrate BRD4 by co-immunocoprecipitation (co-IP) assay to assess whether ultrasound destruction of drug-carrying microbubbles can promote this mechanism. As expected, co-IP results showed that both ARV-825 and ARV-MBs could promote the interaction between BRD4 and CRBN, while ARV-MBs + US treatment could further enhance the binding between them (Fig. 8E). Similarly, the destruction of drug-carrying microbubbles by ultrasound enhanced the ubiquitination of BRD4 induced by ARV-825 (Fig. 8F).

### In vitro and in vivo biosafety

**Fig. 9.**
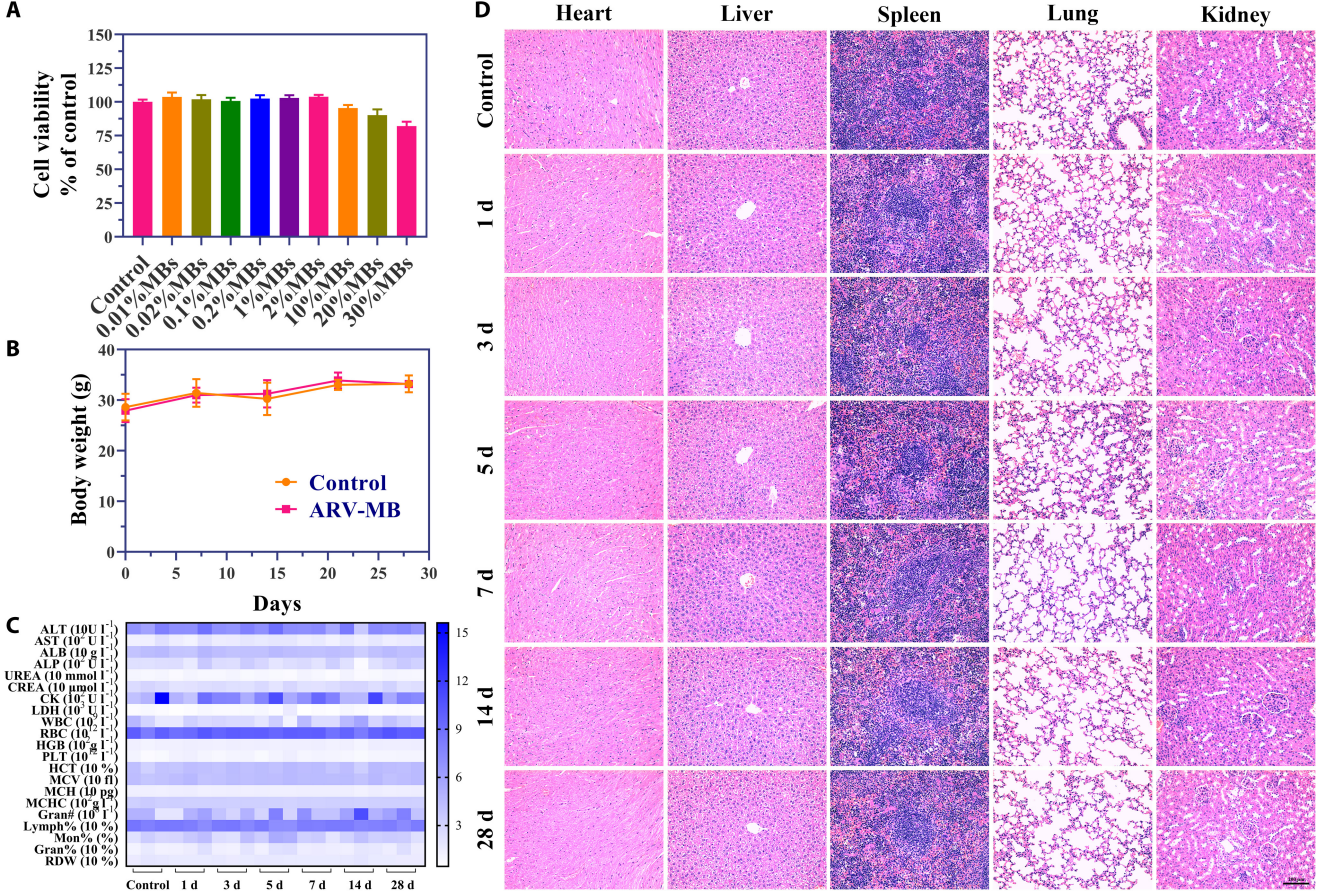
Toxicity assessment in vitro and in vivo. (A) Relative cell viability studies by CCK-8 assay after microbubble treatment at various concentrations for 24 h (*n* = 6). (B) Mice body weight change at different time points after intravenous administration of saline (control) or ARV-MBs (*n* = 5). (C) Serologic biochemical index and blood routine detection of mice at different time points (*n* = 3). (D) H&E staining of the vital organ sections. Scale bar, 200 μm.

The toxicity of microbubbles or ultrasound on cells was detected by CCK-8. The results indicated that different concentrations of unloaded microbubbles or different conditions of ultrasound treated for 24 h had no significant effect on cell survival rate (Fig. 9A and Fig. S3C), indicating that microbubbles and ultrasound treatment alone had good safety for cells. The safety of drug-carrying microbubbles was mainly discussed in mice, because the drug-carrying microbubbles would spontaneously burst if incubated for a very long time.

In vivo biosafety is a key aspect of whether a novel drug preparation can enter the clinic. Therefore, this study preliminarily evaluated the in vivo safety and biocompatibility of ARV-MBs in healthy mice. The results showed that there was no obvious abnormality in behavior and life state during the 1-month observation period after injection of ARV-MBs. There was also no significant difference in body weight between the control and ARV-MBs group during the monitoring (Fig. 9B). At different time points after treatment, the blood routine and blood biochemistry (including markers related to heart, liver, and kidney function) of mice in each group were all within the normal reference range or had no statistical difference with the control group (Fig. 9C). H&E staining of major organs (heart, liver, spleen, lung, and kidney) showed no significant pathological structural changes observed after ARV-MBs injection (Fig. 9D). Specially, since PROTACs have shown liver toxicity in clinical trials, we further conducted the TUNEL assay to determine the extent of any potential hepatotoxic effects. TUNEL staining of liver tissues demonstrated that ARV-MBs did not significantly cause the apoptosis of liver cells during 28 days of observation (Fig. S4). These results fully indicated that ARV-MBs had no obvious biological toxicity in vivo.

## Discussion

PROTAC is a newly emerging technology for targeted posttranslational knockdown of POI, which has been used in various disease especially cancer therapy [32]. The heterobifunctional structure of PROTAC allows dictating the POI for proteolysis via the ubiquitin–proteasome system [33]. Owing to its event-driven mechanism of action, PROTAC offers therapeutic advantages over traditional occupancy-driven inhibitors in terms of low dose, low drug resistance, and “undruggable” protein regulation [34]. As PROTAC-induced protein degradation is a repetitional process, it can potentially have more persistent action than inhibitors or genetic tools, consequently lowering the administration doses [35]. However, in the current studies, the application of PROTAC is challenging due to the poor tumor specificity and unfavorable biodistribution in noncancerous tissues, which might cause potential systemic toxicity [36]. Meanwhile, it suffers from low aqueous solubility and poor permeability [37]. As one of the most promising strategies to enhance drug delivery, UTMD-mediated delivery system has also been seriously limited due to the poor drug loading capacity of microbubbles [28].

In this study, with the properties of PROTAC and UTMD technology, we developed a novel microbubble carrying ARV-825 and showed that it is possible to overcome the disadvantages of PROTAC as well as the low drug loading obstacle of microbubble. We successfully prepared ARV-825-loaded phospholipid microbubbles at a high encapsulation efficiency and loading content. The basic characteristics and imaging performance of microbubbles are not affected after PROTAC loading. In in vivo CEUS imaging study, ARV-MBs produced a visible contrast enhancement for tumor imaging similar to unloaded microbubbles, suggesting that it could realize both guiding and monitoring for tumor therapies. With in vitro CEUS imaging, we validated that the prepared microbubbles could be fragmented when exposed to a certain intensity of ultrasound, indicating the maintenance of ultrasound-responsive ability, which also provided a reference for ultrasound parameters for in vivo therapy. Although strategies such as liposomes conjugation and an inner oil layer addition in the bubble shell can increase the loading content of microbubbles to a certain extent, the payload, however, remains rather limited. Moreover, these methods based on the complex transformation of microbubble structure may lead to some new challenges, such as low yields, instability, and loss of the capability to be imaged and controlled by ultrasound [29], which limited the clinical translation of these strategies. To address the low drug loading efficiency of microbubbles, we present a new strategy by selecting the antitumor drug with a powerful activity (10-hydroxycamptothecin, 10-HCPT) and encapsulating it into microbubble in our previous work [29]. In this study, we provide a better solution by manufacturing microbubbles carrying a newly emerged antitumor drug ARV-825, which not only exhibits powerful activity at a low dosage [38] but also shows persistent and recyclable action on oncoprotein degradation due to its intrinsic catalytic capacity [39]. In addition, the strong lipophilicity of PROTACs makes it conducive to load into the phospholipid shell of microbubbles.

From the drug release and biodistribution profiles, a remarkable drug accumulation was found in the group of ARV-MBs followed by ultrasound exposure but not in ARV-MBs alone. In the presence of ultrasound, the drug-loaded microbubbles were destroyed and fragmented, which were capable of extravasating and accumulating into the tumor tissue and slowly releasing the drug [40,41]. Therefore, encouragingly, the ARV-825 concentration in tumor tissue was markedly elevated by 3.5-fold in the ARV-MBs + US group. Furthermore, the combination of ARV-MBs and ultrasound can increase the cellular uptake of ARV-825, as demonstrated by the increased amount of ARV-825 in MDA-MB-231 cells via RP-HPLC. Compared with the current published targeting strategies with ligand modification or controlled modalities with light or tumor-responsive materials for PROTAC [42,43], our work offers distinct advantages because it solves the obstacles of targeted delivery and cell membrane permeability at the same time. Since PROTACs have entered clinical trials and microbubbles have been used in clinic for years, compared with the complex modification of carriers or drugs, the combination of PROTAC and UTMD is easy to implement and holds great potential for clinical translation. In detail, the strategy we presented on one hand can circumvent the worrying potential off-target toxicity of potent PROTAC by releasing drugs locally in tumor foci through ultrasound destruction of microbubbles, which in turn reduced the administration dosage of drugs and increase the therapeutic window. More importantly, the cavitation effect caused by UTMD can promote the entry of PROTAC into tumor cells and overcome the limitation of poor membrane permeability of PROTAC, leading to enhanced antitumor potency in further study.

For antitumor experiments, the ARV-MBs + US group showed the most efficient tumor inhibition in either 2D cultured cancer cells or 3D tumor spheroids. In in vivo study, a TNBC mouse model was established to evaluate the therapeutic effect of ARV-MBs combined with ultrasound. As we expected, the combination of ARV-MBs and ultrasound resulted in a stronger tumor inhibition in comparison to ARV-MBs alone or ARV-825 injection as a result of the effects described above. The PCNA and TUNEL staining suggested the strongest inhibitory effect on cell proliferation and the presence of largest number of apoptotic cells in the ARV-MBs + US group, respectively. In our previous study, the combination of ultrasound and microbubble may also cause some effects on cellular and capillary levels, leading to a reduction in tumor growth [44], which is consistent with the results in the present study. In addition, the biosafety analysis showed that the ARV-MBs prepared in this study showed no obvious systemic toxicity and good biocompatibility on mice. Mechanically, ARV-MBs combined with ultrasound treatment robustly enhanced the interaction between BRD4 and E3 ligase CRBN, as well as the subsequent ubiquitylation and degradation of BRD4. These may be attributed to that the acoustic pore effect generated by UTMD causes more drugs entering into cells [45], which further provides reliable evidence to the augmented antitumor efficacy of ARV-MBs + US. Therefore, this study provides a novel PROTAC controlled-release method, which does not change the structure of PROTAC itself, avoiding complex structural modification, and preserving the activity of PROTAC to the maximum extent. At the same time, the potent effect of PROTAC also provides the possibility for clinical translation of drug-carrying microbubble-mediated therapy by overcoming the low drug loading obstacles.

However, only one PROTAC (ARV-825) was studied in this study, and PROTACs targeting other tumor therapeutic targets need to be further evaluated. This study only focused on human TNBC cell line MDA-MB-231 and its transplanted tumors, and the antitumor effect of ultrasound destruction of PROTAC-MBs in other types of tumors needs to be further verified. In this study, only short-term treatment observation and degradation effect of BRD4 were conducted in animals at the end of monitoring, and survival observation and long-term effects of ARV-MBs + US were not conducted. These will be the focus of our next study.

In summary, in the current study, a novel clinically translatable PROTAC-loaded microbubbles (ARV-MBs) was successfully constructed for ultrasound-augmented therapeutic efficacy in a TNBC model. We demonstrated that the ARV-MBs combined with ultrasound irradiation promoted tumor delivery and permeability of PROTAC, which led to significant cancer toxicity in vitro and in vivo. Importantly, our strategy caused an enhanced interaction of BRD4 and CRBN, consequently resulting in ubiquitination and degradation of BRD4 protein. The present work highlights the tremendous clinical promise of the newer-generation UTMD-enhanced PROTACs in improving tumor delivery and cellular uptake, which expands the toolbox for targeted protein degradation and UTMD-based cancer therapy, and warrants extensive evaluation in clinical settings.

## Data Availability

Additional data related to this article may be available from the corresponding author on reasonable request.
