# Erratum to “Ultrasound Controllable Release of Proteolysis Targeting Chimeras for Triple-Negative Breast Cancer Treatment”

**DOI:** 10.34133/bmr.0088

**Published:** 2024-10-07

**Authors:** Hongye He, Feng Li, Rui Tang, Nianhong Wu, Ying Zhou, Yuting Cao, Can Wang, Li Wan, Yang Zhou, Hua Zhuang, Pan Li

**Affiliations:** ^1^ Ultrasound Department of the Second Affiliated Hospital of Chongqing Medical University, Chongqing Key Laboratory of Ultrasound Molecular Imaging, Chongqing 400010, China.; ^2^ Department of Hepatobiliary and Pancreatic Surgery, The First Affiliated Hospital of Chongqing Medical University, Chongqing 400016, China.; ^3^ Department of Ultrasound, The Ninth People's Hospital of Chongqing, Chongqing 400700, China.; ^4^ Department of Geriatrics, The Second Affiliated Hospital of Chongqing Medical University, Chongqing 400010, China.; ^5^ Department of Ultrasound, The Third People's Hospital of Chengdu City, The Affiliated Hospital of Southwest Jiaotong University, Chengdu 610014, China.; ^6^ Department of Medical Ultrasound, West China Hospital of Sichuan University, Chengdu 610041, China.

In the Research Article “Ultrasound controllable release of proteolysis targeting chimeras for triple-negative breast cancer treatment”, errors were present in the affiliations for several authors [1]. The corrected affiliations are listed below, and the publisher apologizes for these errors.

• Hongye He: Ultrasound Department of the Second Affiliated Hospital of Chongqing Medical University, Chongqing Key Laboratory of Ultrasound Molecular Imaging, Chongqing 400010, China.
• Ying Zhou: Department of Ultrasound, The Ninth People’s Hospital of Chongqing, Chongqing 400700, China.
• Can Wang: Department of Geriatrics, The Second Affiliated Hospital of Chongqing Medical University, Chongqing 400010, China.
• Yang Zhou: Department of Ultrasound, The Third People’s Hospital of Chengdu City, The Affiliated Hospital of Southwest Jiaotong University, Chengdu 610014, China.
• Hua Zhuang: Department of Medical Ultrasound, West China Hospital of Sichuan University, Chengdu 610041, China.
