# Supplementary material for: Ultrasound Controllable Release of Proteolysis Targeting Chimeras for Triple-Negative Breast Cancer Treatment
Source: Biomater Res. 2024 Aug 13;28:0064. doi: 10.34133/bmr.0064 (PMC11319668; doi:10.34133/bmr.0064)
Supplement: Supplementary 1 — Materials and Methods Figs. S1 to S4 [file bmr.0064.f1.docx]

***Supporting Information for***

**Ultrasound Controllable Release of Proteolysis Targeting Chimeras for Triple Negative Breast Cancer Treatment**

Hongye He^1†^, Feng Li^2†^, Rui Tang^1^, Nianhong Wu^1^, Ying Zhou^3^, Yuting Cao^1^, Can Wang^4^, Li Wan^1^, Yang Zhou^5^, Hua Zhuang^6*^, Pan Li^1*^.

^1^Institute of Ultrasound Imaging & Department of Ultrasound, The Second Affiliated Hospital of Chongqing Medical University, Chongqing Key Laboratory of Ultrasound Molecular Imaging, Chongqing 400010, China.

^2^Department of Hepatobiliary and Pancreatic Surgery, The First Affiliated Hospital of Chongqing Medical University, Chongqing 400016, China.

^3^Department of Ultrasound, The Ninth People's Hospital of Chongqing, Chongqing 400700, China.

^4^Department of geriatrics, The Second Affiliated Hospital of Chongqing Medical University, Chongqing 400010, China.

^5^Department of Ultrasound, The Third People's Hospital of Chengdu City, The Affiliated Hospital of Southwest Jiaotong University, Chengdu 610014, China.

^6^Department of Medical Ultrasound, West China Hospital of Sichuan University, Chengdu 610041, China.

^*^Corresponding authors: Pan Li (lipan@hospital.cqmu.edu.cn), Hua Zhuang (annzhuang@yeah.net).

**Supplemental materials and methods**

**1. Cell lines and reagents**

MDA-MB-231 and HUVEC cells were from the Institute of Ultrasound Imaging of Chongqing Medical University and were cultivated in High glucose Dulbecco’s modified Eagle’s medium (DMEM) containing 10% fetal bovine serum (FBS) and 1% penicillin-streptomycin in an incubator (37℃, 5% CO_2_). 1,2-distearoyl-sn-glycero-3-phosphoethanolamine-N-[methoxy(polyethylene glycol)-2000] (DSPE-mPEG2000) and 1,2-dipalmitoyl-sn-glycero-3-phosphocholine (DPPC) were acquired from Xi’an Ruixi Biological Technology Co., Ltd. (Xi’an, China). ARV-825 was purchased from Selleck (Texas, USA). MG-132 proteasome inhibitor was ordered from Medchemexpress (Shanghai, China). Matrigel was supplied by Corning (New York, USA). Lipofectamine2000 was obtained from Invitrogen (California, USA). DiI and BCA protein quantification kit was obtained from Beyotime (Shanghai, China). RIPA lysis buffer, 5×SDS loading buffer, and dimethyl sulfoxide (DMSO) was purchased from Solarbio (Beijing, China). Phosphate-buffered saline (PBS) was acquired from Servicebio (Wuhan, China). Protein A/G PLUS-Agarose was purchased from Santa Cruz Biotechnology (Texas, USA). All plasmids (pcDNA3.1, Flag-CRBN, HA-Ub) were synthesized by Qingke Biotechnology (Nanjing, China). High glucose DMEM was purchased from Gibco (MA, USA), and FBS was obtained from Biological Industries (CT, USA). Cell counting kit-8 (CCK-8) was purchased from GlpBio (USA). Annexin V-FITC/PI Apoptosis Detection Kit were obtained from Elabscience Biotechnology (Wuhan, China). The anti-rabbit Flag, anti-rabbit BRD4, peroxidase-conjugated anti-mouse IgG, and peroxidase-conjugated anti-rabbit IgG antibodies were acquired from Cell Signaling Technology (MA, USA). The anti-rabbit c-Myc antibody was obtained from Abcam (Cambridge, UK). Anti-mouse HA and Flag antibodies were purchased from ABclonal (Wuhan, China). Anti-mouse β-actin antibody was provided by Proteintech (Wuhan, China). Calcein-AM/PI Double Staining Kit was purchased from Dojindo Laboratories (Kyushu Island, Japan).

**2. Cellular uptake**

MDA-MB-231 cells were placed into a 3.5 cm confocal dish and cultured for 24 h. The cells were treated with DiI-labeled microbubbles (MBs) with or without ultrasonic irradiation (1.0 MHz, 30% duty ratio, 1 W/cm^2^, 30 s). After 0.5, 1, 2, 4 and 6 h, the cells were harvested for further analysis. For fluorescence microscope observation, the cells were washed with PBS and fixed with 4% paraformaldehyde for 15 min. Then DAPI nuclear dye was added and incubated for another 5-10 min. Finally, the cells were photographed under a confocal laser scanning microscopy (CLSM). For flow cytometer detection, the cells after the same treatment were rinsed with PBS, then single-cell suspension was prepared by digestion with trypsin. The suspension was determined via a BD LSRFortessa flow cytometer (BD, USA).

**3. 3D spheroid penetration**

MDA-MB-231 cells were cultured in ultra-low adhesion 96-well plates for 7-10 days with medium changed every 2 days to form 3D spheroid. The DiI-labeled MBs were added with or without ultrasound irradiation (1.0 MHz, 30% duty ratio, 1 W/cm^2^, 30 s). After incubation at 37℃ for 2 h, the cells were washed with PBS and fixed with 4% paraformaldehyde for 15 min. DAPI nuclear dye was added and incubated for 30 min. Then the cells were washed with PBS, and photographed via CLSM.

**4. *In vivo* and *ex vivo* biodistribution studies**

Tumor bearing mice were randomly divided into two groups (n=3). The DiR-labeled ARV-MBs (containing ARV-825 3.5 mg/kg, 200 μL) was *i.v.* injected with or without ultrasound (1.0 MHz, 30% duty ratio, 2.0 W/cm^2^, 3 min) after injection. Fluorescence images were collected before treatment, 1 h, 2 h, 4 h, 8 h, 24 h and 48 h after treatment using an IVIS fluorescence imaging system. Mice were sacrificed at 48 h, and important organs (heart, liver, spleen, lung, and kidney) and tumors were harvested and also imaged. The fluorescence intensity was analyzed using IndiGo software. Furthermore, the cell nucleus was dyed with DAPI and the fluorescence distribution in the tumor tissue was observed and photographed.

**5. *In vitro* and *in vivo* contrast-enhanced ultrasound (CEUS)**

The microbubbles were diluted with normal saline and added into the agarose gel mold. The contrast ultrasound imaging was carried out on a photoacoustic machine (VivoLaser). The microbubbles irradiated by ultrasound under different conditions were also imaged.

Mice with tumor were anesthetized by intraperitoneal injection of 1% sodium pentobarbital and fixed on the imaging console. The CEUS was carried out on a photoacoustic machine. The microbubbles were injected through the tail vein at a dose of 0.2 mL. The ultrasound imaging at tumor site was stored. The Vevo LAB software was used to analyze the time-intensity curve of ROI.

**6. Cell viability and apoptosis assay**

HUVEC or MDA-MB-231 cells (5×10^3^ cells/well) were seeded into 96-well plates. After 24 h, cells were treated with ARV-825 at different concentrations and incubated for another 24 h or 48 h. The cell viability was then examined by CCK-8 assay. MDA-MB-231 cells were seeded into 96-well plates, confocal dishes or 6-well plates respectively, and were grouped as: Control, US, MBs+US, ARV-825 (2 μM), ARV-MBs (ARV-825: 2 μM), ARV-MBs (ARV-825: 2 μM) +US. The ultrasonic irradiation group was treated with ultrasound (1.0 MHz, 1.0 W/cm^2^, 30% duty ratio, 30 s) immediately after adding the corresponding reagent. After 24 h, each well was washed with PBS. The cell viability was tested by CCK-8 or Live/Dead staining assay according to the manufacture’s instruction. For flow cytometry, cells after treatment were collected and re-suspended with 200 μL of Annexin V/PI dyes, incubated for 15 min, followed by analysis on CytoFLEX (Beckman Coulter Life Sciences). MDA-MB-231 cells (1.5×10^3^ cells/well) were inoculated into ultra-low adhesion 96-well plates. After 7 days, the cells were grouped as above. After 24 h of treatment, the Live/Dead (Calcein-AM 1 μM, PI 3 μM) dyes were added and incubated for 3 h. After carefully washing with PBS, the cells were observed and photographed under CLSM.

**7. *In vitro* and *in vivo* biosafety**

The cells were seeded into 96-well plates and irradiated with different ultrasound (1, 2 and 3 W/cm^2^ for 15, 30 and 60 s, respectively). CCK-8 detection was performed after 24 h of treatment. Moreover, MBs was diluted into different concentrations and added in 96-well plates (100 μL/ well). After 24 h, the cell viability was detected by CCK-8 kit also.

Healthy Kunming mice (6-8 weeks) were provided by Laboratory Animal Center of Chongqing Medical University. The mice were randomly divided into 7 groups (n=5). The control group was *i.v.* injected with 200 μL of saline, and the other groups with ARV-MBs (ARV-825: 3.5 mg/kg). Mice were sacrificed at 1 d, 3 d, 5 d, 7 d, 14 d and 28 d after injection. Serum and plasma were collected for serological biochemistry analysis and blood routine, respectively. Major organs were harvested for hematoxylin and eosin (H&E) staining and livers for TUNEL staining.

**8. Western blot**

Cells were lysed by RIPA lysis buffer supplemented with 1% phenylmethylsulfonyl fluoride (PMSF). After centrifugation at 12000 rpm for 15 min, the supernatant was collected. Protein concentration was determined according to the instructions of BCA protein assay kit. Immunoblot analysis was conducted following standard protocol. In addition, the tumors of mice were collected and homogenized with RIPA lysis buffer containing 1% PMSF. Then the samples were lysed on ice for 30 min, and centrifuged at 12000 rpm for 15 min at 4℃. The supernatant was collected and subjected for immunoblotting by standard protocol.

**Supplemental figures**


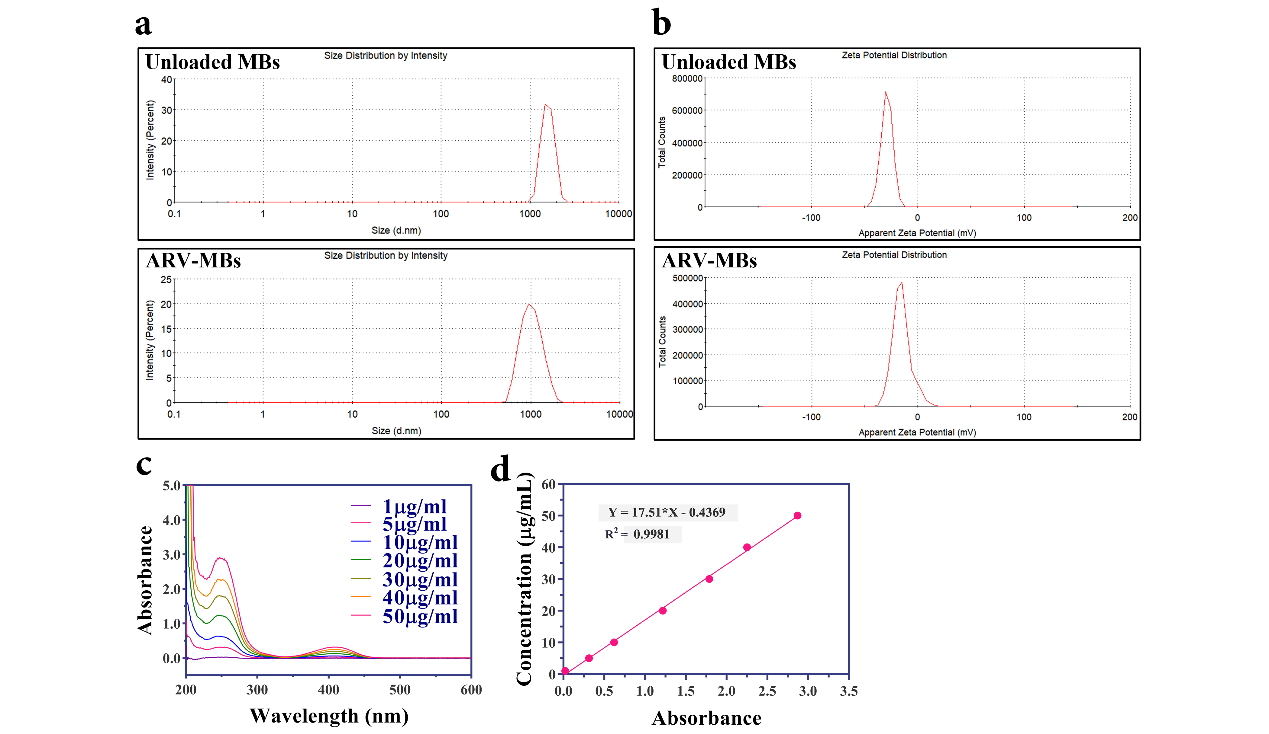


**Fig. S1.** The characterization of ARV-MBs. (**a**) Size distribution of unloaded MBs and ARV-MBs. (**b**) Zeta potential of unloaded MBs and ARV-MBs. (**c, d**) The UV-Vis absorption of ARV-825 at different concentrations (c) and its corresponding standard curve at 250 nm (d).


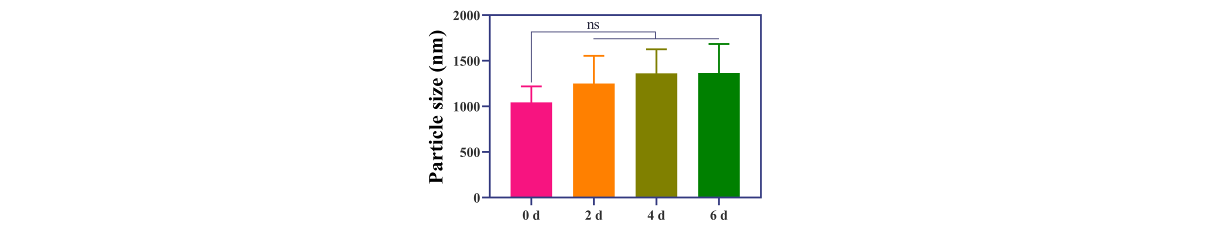


**Fig. S2.** The size stability of ARV-MBs during storage at 4℃ (ns: *p* > 0.05).


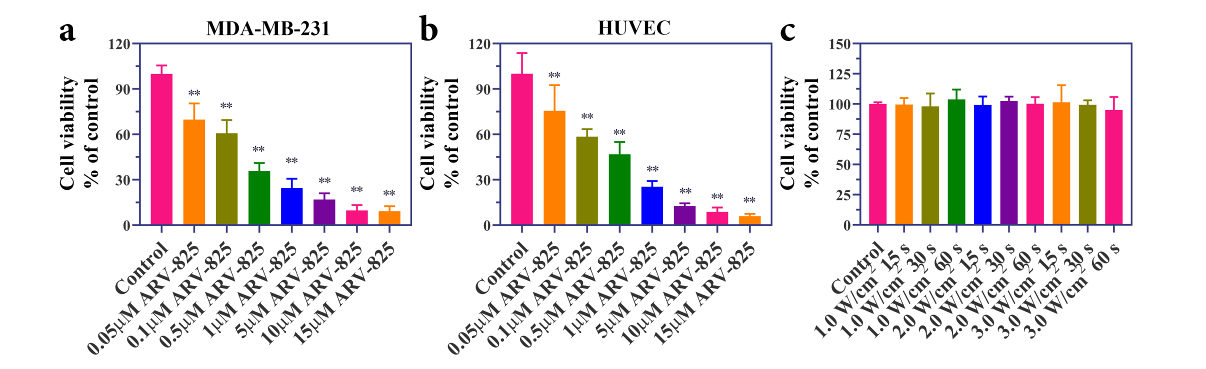


**Fig. S3.** Toxicity assessment *in vitro*. (**a, b**) The cell viability assays using CCK-8 reagent were conducted in MDA-MB-231 (a, n=6) and HUVEC cells (b, n=5) incubated with ARV-825 at different concentration for 72 h. ***p* < 0.01. (**c**) Relative cell viability studies by CCK-8 assay after ultrasound irradiation at different conditions for 24 h (n=4).


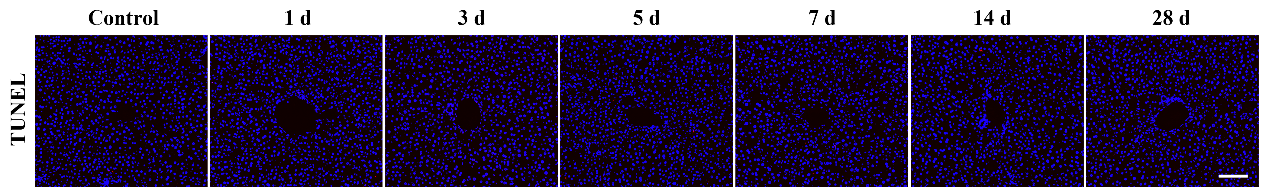


**Fig. S4**. Hepatotoxic effects of ARV-MBs as determined by TUNEL staining. Scale bar is 100 μm.
